# A study investigating how the albumin-globulin ratio relates to depression risk within U.S. adults: a cross-sectional analysis

**DOI:** 10.3389/fnut.2024.1453044

**Published:** 2024-10-03

**Authors:** Qi Xu, Jiale Wang, Hanzhi Li, Xiaohui Chen

**Affiliations:** ^1^Department of Breast and Thyroid Surgery, The First Affiliated Hospital of Nanyang Medical College, Nanyang, China; ^2^Department of Breast Surgery, Harbin Medical University Cancer Hospital, Harbin, China; ^3^Department of Internal Medicine, The Affiliated Jiangning Hospital of Nanjing Medical University, Nanjing, China; ^4^Department of Breast and Thyroid Surgery, The Second Affiliated Hospital of Guilin Medical University, Guilin, China

**Keywords:** National Health and Nutrition Examination Survey, depression, albumin-globulin ratio, chronic inflammation, adults

## Abstract

**Background:**

The relationship between the albumin-to-globulin ratio (AGR) and depression is not well understood. This analysis aims to investigate the relationship between AGR in conjunction with depression in U.S. adults.

**Methods:**

This study analyzed information from 31,363 individuals collected by NHANES during the years 2005 to 2018. The PHQ-9 scale was employed to gauge depression, where a score of 10 or above signified depression. Weighted multivariable logistic modeling along with smooth curve fitting were applied to explore the AGR-depression connection. To confirm our findings, we carried out sensitivity analyses, subgroup analyses, and interaction tests.

**Results:**

After adjusting for confounding variables, a higher AGR is associated with a lower risk of depression (OR = 0.61, 95% CI: 0.47–0.79). Dividing AGR into quartiles revealed that participants in the highest quartile (Q4) of AGR had a markedly lower risk of depression than those in the lowest quartile (Q1) (OR = 0.64, 95% CI: 0.53–0.77). Using smooth curve fitting, we suggested a possible linear inverse association connecting AGR with depression. Further subgroup and sensitivity analyses supported these findings, although factors such as diabetes and hypertension might influence the relationship.

**Conclusion:**

Our findings indicate that elevated AGR levels correlate with a lower risk of depression. The findings suggest AGR as a potential biomarker for depression screening and prevention. Further studies are required to determine causality and clarify the mechanisms between AGR and depression.

## 1 Introduction

Depression is a significant mental health issue that impacts approximately 350 million individuals around the globe, according to the World Health Organization (WHO) (1). It is expected to become the top cause of global disease burden by 2030 (2). Over 20% of individuals worldwide will experience depression at least once in their lifetime (3).

The disorder is characterized by persistently low mood, changes in cognitive function, and diminished interest in daily activities (4). Despite various treatment options, pharmacotherapy is the primary choice due to its convenience and relative safety (5). Nevertheless, common antidepressants, such as selective serotonin reuptake inhibitors (SSRIs) and tricyclic antidepressants (TCAs), can lead to weight gain, insomnia, and sexual dysfunction, which affect patients’ quality of life and their adherence to treatment. This situation makes it particularly important to find new biomarkers and therapeutic targets in the prevention and treatment of depression.

In recent years, researchers have paid increasing attention to the role of inflammation and nutritional status in the pathogenesis of depression. Albumin, the major plasma protein produced by the liver, is crucial for regulating osmotic pressure and transporting various substances, including hormones, drugs, and fatty acids. Albumin also reflects nutritional status and inflammatory responses (6). Moreover, globulins, which are involved in immune and inflammatory processes, increase during inflammation. The albumin-globulin ratio (AGR) combines the advantages of these two proteins and has been widely studied in a variety of diseases, including malignant tumors, acute ischemic stroke, chronic heart failure, etc. (7–13).

Although studies have shown that patients suffering from depression show significantly decreased albumin levels, while their α1-globulin, α2-globulin, and β-globulin levels are notably higher (14). However, research on the relationship between AGR and depression is very limited. To date, no systematic study has clearly explored the link between AGR and the risk of depression. Considering the complex etiology of depression and the limitations of existing treatments, clarifying the relationship between AGR and depression may provide new ideas and tools for the prevention and early intervention of depression.

Therefore, this study aims to systematically evaluate the association between albumin-globulin ratio and the risk of depression in American adults using the National Health and Nutrition Examination Survey (NHANES) data. This study not only fills the gap in the existing literature, but also provides an important basis for the development of depression screening and intervention strategies based on AGR in the future.

## 2 Materials and methods

### 2.1 Study design and participants

Conducted by the National Center for Health Statistics (NCHS), the NHANES database is a nationwide research project that collects data on the health and nutritional conditions of Americans (15). The project utilized an advanced stratified, multistage, complex probability sampling method to ensure participant representativeness (16). All participants were thoroughly briefed on the study and signed informed consent forms before data collection commenced. The NCHS Ethical Review Committee reviewed and approved all research protocols. Furthermore, the official NHANES website provides open access to the datasets, as well as the related documentation and protocols.

**Figure 1:**
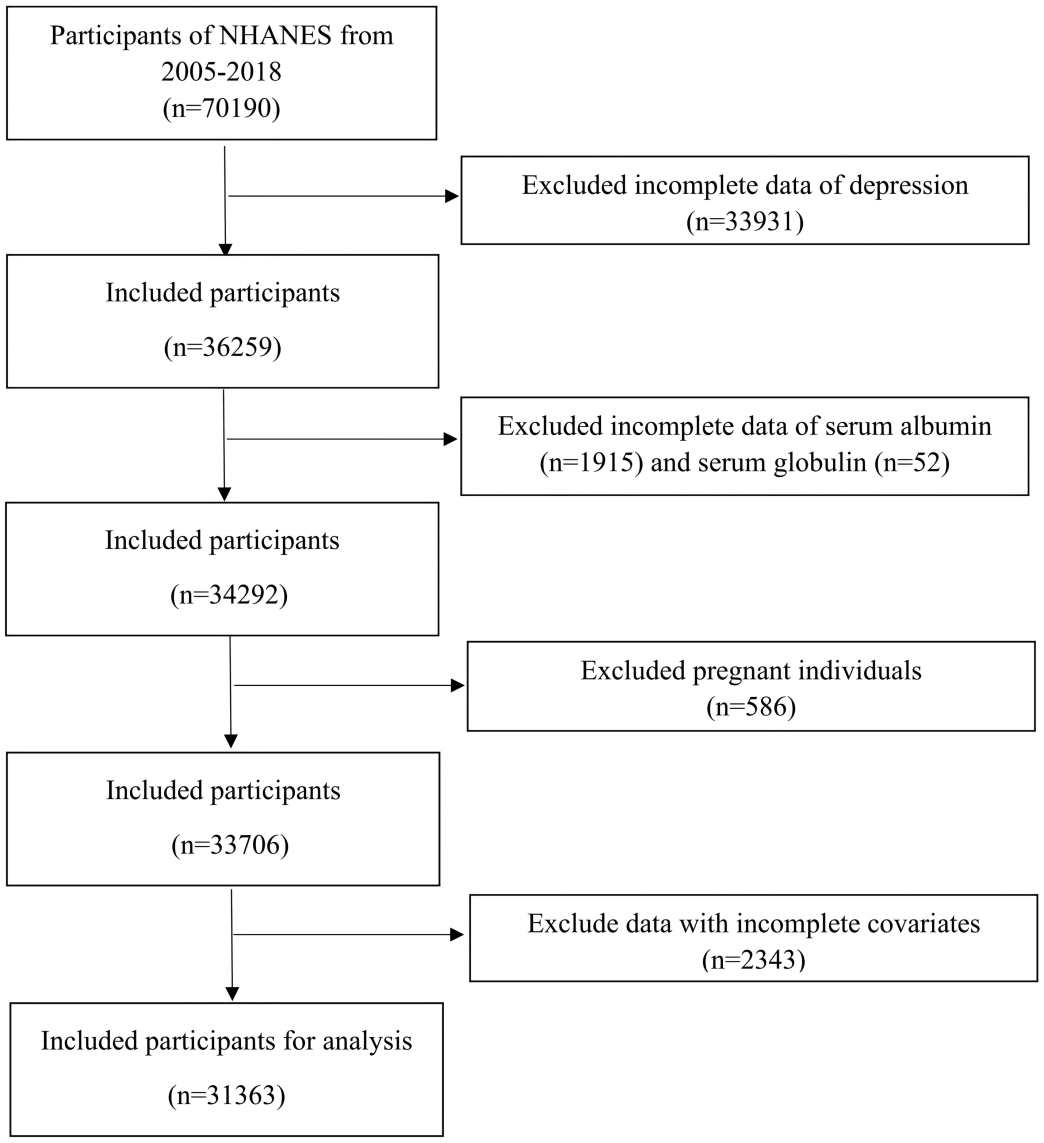
Flowchart depicting the procedure for sample selection from NHANES 2005–2018.

For our study, we sourced data from seven NHANES survey cycles covering the years 2005 to 2018. These specific cycles were selected due to the availability of complete data for the Patient Health Questionnaire-9 (PHQ-9), albumin, and globulin. Participants were excluded if they had: (1) no PHQ-9 data; (2) no albumin and globulin data; (3) pregnancy; (4) missing information on covariates including education level, marital status, BMI, smoking status, cholesterol, triglycerides, CVD, hypertension, and cancer. Ultimately, we included 31,363 participants in the study. Figure 1 provides a comprehensive overview of the screening process.

### 2.2 Assessment of depression

To assess depression, we utilized the PHQ-9 questionnaire, which consists of nine questions based on depressive symptoms, to evaluate their frequency over the past 2 weeks (17). Each response is scored from 0 to 3, indicating different levels of symptom frequency. These scores are summed, resulting in a total score ranging from 0 to 27, which determines the severity of depressive symptoms (18). We classified participants as having depression if their PHQ-9 score was 10 or higher, and as not having depression if their score was below 10. This threshold is highly effective, demonstrating 88% specificity and sensitivity in detecting depression (19).

### 2.3 Albumin, globulin, and AGR

The albumin concentration was determined by measuring the absorbance change at 600 nm after binding with bromocresol purple, the total protein concentration was determined by monitoring the chelate formation rate at 545 nm using the biuret method, and the globulin concentration was calculated by subtracting the albumin concentration from the total protein to ensure accurate results (20). Both serum albumin (g/dL) and serum globulin (g/dL) levels were measured utilizing a Beckman UniCel^®^ DxC800 Synchron analyzer. Based on these parameters, the AGR can be calculated by dividing the albumin concentration (g/dL) by the globulin concentration (g/dL).

### 2.4 Covariates

Our study considered several potential covariates, including age, gender (male and female), race/ethnicity (Mexican American, other Hispanic, Non-Hispanic White, Non-Hispanic Black, and other), education level (less than high school, high school or GED, college or higher), marital status (married/living with partner, separated/divorced/widowed, never married), and BMI categories (<25.0, 25.0–29.9, and ≥30.0 kg/m^2^). Smoking status was divided into never smoked (fewer than 100 cigarettes lifetime), former smoker (more than 100 cigarettes lifetime, but not currently smoking), and current smoker (more than 100 cigarettes lifetime and currently smoking occasionally or daily). Hypertension was defined by a history of hypertension, current antihypertensive treatment, or systolic blood pressure ≥ 140 mmHg and/or diastolic blood pressure ≥ 90 mmHg without medication. Diabetes criteria included a history of diabetes, current use of oral hypoglycemic agents or insulin, fasting plasma glucose ≥126 mg/dL, 2-h plasma glucose ≥200 mg/dL post-OGTT, or HbA1c ≥6.5%. Participants diagnosed with congestive heart failure, coronary artery disease, angina, myocardial infarction, or stroke were classified as having CVD. Cancer status was determined based on physician diagnosis. Laboratory measurements included blood urea nitrogen, cholesterol, total protein, triglycerides, uric acid, and creatinine.

### 2.5 Statistical analysis

This study incorporated sample weights into all statistical analyses, acknowledging the complexity of the survey design and adhering to CDC guidelines for data processing. Baseline characteristics were categorized into two sets: one based on the presence or absence of depression and another by AGR quartiles (Q1 < 1.29, Q21.29–1.46, Q3 1.47–1.66, Q4 > 1.66). For continuous variables, linear regression was utilized, and chi-square tests were used for categorical variables.

To investigate the relationship between AGR and depression, we initially used multivariate logistic regression. Following this, a multivariate linear regression model was utilized to analyze the link between AGR and PHQ-9 scores. AGR was then transformed from a continuous variable into quartiles to conduct trend tests. Furthermore, a smoothing curve fitting method was used to reveal the trend in the link between AGR and depression.

To explore potential differential associations between AGR and depression in specific populations, we performed subgroup and interaction analyses. Furthermore, we conducted sensitivity analyses: one excluding patients taking antidepressant medication, and another using unweighted raw data for analysis. Finally, we performed regression analyses on all items of the PHQ-9.

We utilized R software (version 4.2) and EmpowerStats software (version 4.1) to perform all statistical analyses. A two-tailed *p*-value of less than 0.05 was considered indicative of statistical significance.

## 3 Results

### 3.1 Baseline characteristics

A total of 31,363 participants were enrolled in this study, comprising 15,661 men and 15,702 women, with a mean age of 47.71 years. A summary of the baseline characteristics of the participants is presented in Table 1. Notably, 8.78% were identified as having depression.

**Table 1:** Baseline characteristics of participants with and without depression in NHANES 2005–2018, weighted.

Characteristics	Total	No depression	Depression	*p-*value
Number of participants	31,363	28,608	2,755	
Age, years	47.71 ± 16.82	47.78 ± 16.91	46.92 ± 15.70	0.016
Sex, *n* (%)				<0.001
Male	15,661 (49.32)	14,646 (50.42)	1,015 (36.03)	
Female	15,702 (50.68)	13,962 (49.58)	1740 (63.97)	
Race/ethnicity, *n* (%)				<0.001
Mexican American	4,929 (8.34)	4,513 (8.37)	416 (7.96)	
Other Hispanic	3,015 (5.42)	2,654 (5.23)	361 (7.73)	
Non-Hispanic White	13,662 (68.71)	12,491 (69.10)	1,171 (63.96)	
Non-Hispanic Black	6,477 (10.41)	5,874 (10.18)	603 (13.19)	
Other races	3,280 (7.12)	3,076 (7.11)	204 (7.17)	
Education level, *n* (%)				<0.001
Less than high school	7,530 (15.31)	6,563 (14.52)	967 (24.91)	
High school or GED	7,241 (23.34)	6,572 (23.05)	669 (26.84)	
College or above	16,592 (61.34)	15,473 (62.43)	1,119 (48.26)	
Marital status, *n* (%)				<0.001
Never married	5,593 (17.62)	5,029 (17.36)	564 (20.69)	
Married/living with partner	18,784 (63.91)	17,529 (65.19)	1,255 (48.49)	
Separated/divorced/widowed	6,986 (18.47)	6,050 (17.45)	936 (30.82)	
BMI (kg/m^2^), *n* (%)				<0.001
<25.0	8,848 (29.46)	8,193 (29.73)	655 (26.25)	
25.0–29.9	10,436 (33.04)	9,713 (33.62)	723 (25.97)	
≥30	12,079 (37.50)	10,702 (36.65)	1,377 (47.78)	
Smoking status, *n* (%)				<0.001
Never	17,141 (54.57)	16,033 (55.94)	1,108 (38.07)	
Former	7,737 (25.23)	7,107 (25.45)	630 (22.61)	
Current	6,485 (20.19)	5,468 (18.61)	1,017 (39.33)	
Diabetes, *n* (%)				<0.001
Yes	4,081 (9.56)	3,537 (9.12)	544 (14.85)	
No	25,436 (84.55)	23,450 (85.20)	1986 (76.71)	
Borderline	1846 (5.89)	1,621 (5.68)	225 (8.44)	
Hypertension, *n* (%)				<0.001
Yes	14,083 (39.67)	12,594 (38.86)	1,489 (49.46)	
No	17,280 (60.33)	16,014 (61.14)	1,266 (50.54)	
CVD, *n* (%)				<0.001
Yes	2,680 (6.83)	2,265 (6.35)	415 (12.52)	
No	28,683 (93.17)	26,343 (93.65)	2,340 (87.48)	
Cancer, *n* (%)				0.014
Yes	2,993 (10.16)	2,683 (10.04)	310 (11.61)	
No	28,370 (89.84)	25,925 (89.96)	2,445 (88.39)	
PHQ-9 scores	3.01 ± 4.05	2.09 ± 2.37	14.04 ± 3.80	<0.001
AGR	1.55 ± 0.30	1.55 ± 0.30	1.47 ± 0.29	<0.001
Albumin, g/dL	4.26 ± 0.33	4.27 ± 0.33	4.18 ± 0.36	<0.001
Globulin, g/dL	2.83 ± 0.43	2.83 ± 0.43	2.92 ± 0.46	<0.001
Blood urea nitrogen mg/dL	13.66 ± 5.36	13.73 ± 5.30	12.83 ± 5.96	<0.001
Cholesterol, mg/dL	194.52 ± 41.23	194.40 ± 40.92	195.96 ± 44.79	0.074
Total protein, g/dL	7.10 ± 0.45	7.10 ± 0.44	7.10 ± 0.47	0.899
Triglycerides, mg/dL	152.22 ± 120.44	151.07 ± 120.10	166.10 ± 123.63	<0.001
Uric acid, mg/dL	5.44 ± 1.40	5.45 ± 1.40	5.32 ± 1.43	<0.001
Creatinine, mg/dL	0.89 ± 0.36	0.90 ± 0.33	0.88 ± 0.58	0.143

Mean ± SD for weighted continuous variables; % for weighted categorical variables. BMI, body mass index; CVD, cardiovascular disease; PHQ-9, patient health questionnaire 9; AGR, albumin to globulin ratio.

When compared to non-depressed participants, those with depression were more likely to be female, Non-Hispanic White, possess higher educational attainment, be married or cohabiting, and present higher rates of obesity, smoking, and chronic diseases such as hypertension, diabetes, cardiovascular diseases, and cancer (all *p* < 0.05). Moreover, individuals with depression exhibited lower levels of blood urea nitrogen and uric acid, alongside elevated triglyceride levels (all *p* < 0.001). The baseline characteristics, categorized by AGR, are provided in Supplementary Table S1.

### 3.2 Association between AGR and depression

Table 2 presents the association between AGR and depression, demonstrating that a higher AGR is significantly associated with a lower risk of depression across all models (*p* < 0.01). Adjusting for potential confounders, each unit rises in AGR corresponded to a 39% lower chance of depression (OR = 0.61, 95% CI: 0.47, 0.79). When analyzing AGR in quartiles, the association remained significant (trend *p* < 0.001). Individuals in the highest AGR quartile (Q4) demonstrated a 36% lower risk of depression relative to those in the lowest quartile (Q1) (OR = 0.64, 95% CI: 0.53, 0.77).

**Table 2:** Association between AGR and depression (logistic regression model).

Characteristics	Model 1		Model 2		Model 3	
	OR (95%CI)	*p-*value	OR (95%CI)	*p-*value	OR (95%CI)	*p-*value
Continuous	0.38 (0.31, 0.47)	<0.001	0.47 (0.37, 0.59)	<0.001	0.61 (0.47, 0.79)	<0.001
AGR quartiles						
Q1	1 (ref)		1 (ref)		1 (ref)	
Q2	0.72 (0.63, 0.83)	<0.001	0.76 (0.66, 0.87)	<0.001	0.84 (0.72, 0.98)	0.022
Q3	0.59 (0.52, 0.69)	<0.001	0.66 (0.57, 0.77)	<0.001	0.75 (0.63, 0.88)	0.001
Q4	0.45 (0.39, 0.52)	<0.001	0.53 (0.45, 0.62)	<0.001	0.64 (0.53, 0.77)	<0.001
*p* for trend		<0.001		<0.001		<0.001

Model 1: no covariates were adjusted. Model 2: adjusted for age, sex, and race/ethnic category. Model 3: adjusted for age, sex, race/ethnicity, education level, marital status, BMI, smoking status, diabetes, hypertension, CVD, cancer, blood urea nitrogen, cholesterol, total protein, triglycerides, uric acid, creatinine. BMI, body mass index; CVD, cardiovascular disease; PHQ-9, Patient Health Questionnaire 9; AGR, albumin to globulin ratio. AGR quartiles: Q1: <1.29; Q2: 1.29–1.46; Q3: 1.47–1.66; Q4: >1.66.

**Figure 2:**
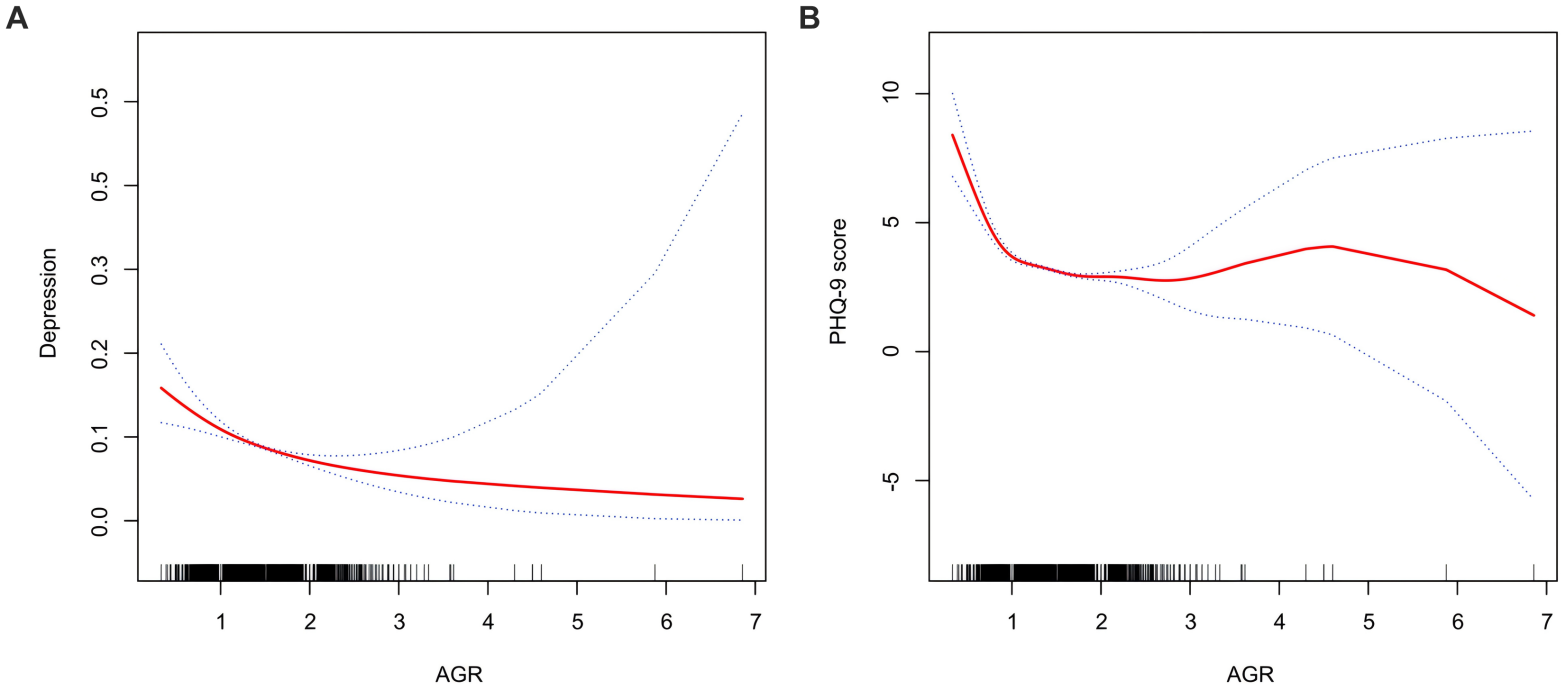
Smooth curve of the trend of the relationship between AGR and depression. The solid red line represents the smooth curve fit between variables. Blue bands represent the 95% of confidence interval from the fit. **(A)** AGR and depression. **(B)** AGR and PHQ-9 score.

Furthermore, the linear regression analysis between AGR and PHQ-9 scores reinforced this finding, showing that a higher AGR is associated with a lower risk of depression (Table 3). The smoothed fitting curve (Figure 2) also revealed a similar trend, corroborating the linear negative relationship between AGR levels and depression, and a non-linear negative association with PHQ-9 scores. These results are consistent with the previously mentioned regression analysis.

**Table 3:** Association between AGR and PHQ-9 scores (linear regression model).

Characteristics	Model 1		Model 2		Model 3	
	β (95%CI)	*p-*value	β (95%CI)	*p-*value	β (95%CI)	*p*-value
Continuous	−1.25(−1.45, −1.05)	<0.001	−0.98(−1.19, −0.76)	<0.001	−0.61(−0.86, −0.37)	<0.001
AGR quartiles						
Q1	0 (ref)		0 (ref)		0 (ref)	
Q2	−0.52(−0.71, −0.34)	<0.001	−0.46(−0.65, −0.28)	<0.001	−0.31(−0.49, −0.13)	0.001
Q3	−0.86(−1.04, −0.68)	<0.001	−0.72(−0.90, −0.53)	<0.001	−0.52(−0.71, −0.33)	<0.001
Q4	−1.16(−1.32, −0.99)	<0.001	−0.95(−1.13, −0.77)	<0.001	−0.63(−0.83, −0.42)	<0.001
*p* for trend		<0.001		<0.001		<0.001

Model 1: no covariates were adjusted. Model 2: adjusted for age, sex, and race/ethnic category. Model 3: adjusted for age, sex, race/ethnicity, education level, marital status, BMI, smoking status, diabetes, hypertension, CVD, cancer, blood urea nitrogen, cholesterol, total protein, triglycerides, uric acid, creatinine. BMI, body mass index; CVD, cardiovascular disease; PHQ-9, Patient Health Questionnaire 9; AGR, albumin to globulin ratio. AGR quartiles: Q1: <1.29; Q2: 1.29–1.46; Q3: 1.47–1.66; Q4: >1.66.

### 3.3 Subgroup analyses

To enhance the validation of the robustness and accuracy of the conclusions concerning AGR and depression among the general adult population in the United States and to identify potential variations within specific subgroups, we conducted subgroup analyses and interaction tests. The stratification factors included age, gender, race, marital status, BMI, smoking status, presence of diabetes, and hypertension.

The findings indicated that the inverse relationship between AGR and depression persisted in most subgroups (Table 4). Interaction tests further revealed that diabetes and hypertension significantly influenced the relationship between AGR and depression (interaction *p* < 0.05). Specifically, among individuals with diabetes, every unit rises in AGR correlated with a 54% decrease in the risk of depression (OR = 0.46, 95% CI: 0.32–0.65). Similarly, among those with hypertension, each incremental increase in AGR led to a 40% drop in depression risk (OR = 0.60, 95% CI: 0.48–0.74). These findings suggest that diabetes and hypertension may significantly modulate the association between AGR and depressive symptoms.

**Table 4:** Subgroup analysis for the association between AGR and depression.

Characteristics	OR (95%CI)	*p-*value	*p* for interaction
Age (years), *n* (%)			0.859
<60	0.65 (0.53, 0.80)	<0.001	
≥60	0.63 (0.48, 0.84)	0.001	
Sex, *n* (%)			0.118
Male	0.55 (0.43, 0.71)	<0.001	
Female	0.70 (0.57, 0.88)	<0.001	
Race/ethnicity, *n* (%)			0.675
Mexican American	0.47 (0.31, 0.73)	<0.001	
Other Hispanic	0.46 (0.30, 0.72)	<0.001	
Non-Hispanic White	0.49 (0.39, 0.62)	<0.001	
Non-Hispanic Black	0.50 (0.35, 0.71)	<0.001	
Other races	0.75 (0.43, 1.28)	0.289	
Marital status, *n* (%)			0.058
Never married	0.60 (0.44, 0.83)	0.002	
Married/living with partner	0.42 (0.33, 0.52)	<0.001	
Separated/divorced/widowed	0.58 (0.44, 0.77)	<0.001	
BMI (kg/m^2^), *n* (%)			0.429
<25.0	0.59 (0.44, 0.79)	<0.001	
25.0–29.9	0.47 (0.35, 0.63)	<0.001	
≥30.0	0.47 (0.37, 0.60)	<0.001	
Smoking status, *n* (%)			0.888
Never	0.49 (0.38, 0.62)	<0.001	
Former	0.49 (0.36, 0.67)	<0.001	
Current	0.53 (0.41, 0.68)	<0.001	
Diabetes, *n* (%)			0.027
Yes	0.46 (0.32, 0.65)	<0.001	
No	0.48 (0.39, 0.58)	<0.001	
Borderline	1.01 (0.59, 1.72)	0.970	
Hypertension, *n* (%)			0.014
Yes	0.60 (0.48, 0.74)	<0.001	
No	0.41 (0.32, 0.52)	<0.001	

### 3.4 Sensitivity analyses

The sensitivity analysis results are comprehensively detailed in Table 5. Excluding individuals on antidepressants, there was a marked reduction in depression risk in Q4 relative to Q1 (OR: 0.66, 95% CI: 0.54, 0.80). The unweighted logistic regression analysis confirmed this trend, also showing a decreased depression risk in Q4 as opposed to Q1 (OR: 0.76, 95% CI: 0.67, 0.87). Both trend tests yielded statistically significant results (trend *p* < 0.01). Additionally, an interesting pattern emerged from the data in Table 6: participants with higher AGR levels scored significantly lower on questions 1, 2, 4, and 5 of the PHQ-9 depression scale, indicating a better overall mental health status.

**Table 5:** Sensitivity analyses.

Characteristics	Model 1		Model 2		Model 3	
	OR (95%CI)	*p-*value	OR (95%CI)	*p-*value	OR (95%CI)	*p-*value
Excluding participants take antidepressants, weighted (*n* = 30,387)						
Continuous	0.39 (0.32, 0.49)	<0.001	0.50 (0.39, 0.64)	<0.001	0.65 (0.49, 0.85)	0.002
AGR quartiles						
Q1	1 (ref)		1 (ref)		1 (ref)	
Q2	0.72 (0.62, 0.83)	<0.001	0.76 (0.65, 0.88)	<0.001	0.84 (0.71, 0.98)	0.026
Q3	0.60 (0.51, 0.70)	<0.001	0.67 (0.57, 0.79)	<0.001	0.75 (0.63, 0.90)	0.001
Q4	0.46 (0.39, 0.54)	<0.001	0.55 (0.46, 0.65)	<0.001	0.66 (0.54, 0.80)	<0.001
*p* for trend		<0.001		<0.001		<0.001
Unweighted (*n* = 31,363)						
Continuous	0.45 (0.39, 0.52)	<0.001	0.50 (0.43, 0.58)	<0.001	0.64 (0.53, 0.76)	<0.001
AGR quartiles						
Q1	1 (ref)		1 (ref)		1 (ref)	
Q2	0.83 (0.75, 0.93)	0.001	0.86 (0.77, 0.95)	0.005	0.94 (0.84, 1.06)	0.311
Q3	0.68 (0.61, 0.76)	<0.001	0.72 (0.65, 0.81)	<0.001	0.83 (0.73, 0.94)	0.002
Q4	0.57 (0.51, 0.64)	<0.001	0.63 (0.56, 0.71)	<0.001	0.76 (0.67, 0.87)	<0.001
*p* for trend		<0.001		<0.001		<0.001

Model 1: no covariates were adjusted. Model 2: adjust for age, sex and race/ethnicity category. Model 3: adjust for age, sex, race/ethnicity, education level, marital status, BMI, smoking status, diabetes, hypertension, CVD, cancer, blood urea nitrogen, cholesterol, total protein, triglycerides, uric acid, creatinine. BMI, body mass index; CVD, cardiovascular disease; PHQ-9, patient health questionnaire 9; AGR, albumin to globulin ratio. AGR quartiles: Q1: <1.29; Q2: 1.29–1.46; Q3: 1.47–1.66; Q4: >1.66.

**Table 6:** Association between serum AGR and each question on the PHQ-9 depression scale.

PHQ-9 depression scale	Model 1	Model 2		Model 3		
	OR (95%CI)	*p-*value	OR (95%CI)	*p-*value	OR (95%CI)	*p-*value
1. Have little interest in doing things	0.62 (0.54, 0.70)	<0.001	0.72 (0.63, 0.83)	<0.001	0.82 (0.70, 0.95)	0.009
2. Feeling down, depressed, or hopeless	0.60 (0.53, 0.68)	<0.001	0.71 (0.61, 0.81)	<0.001	0.80 (0.69, 0.94)	0.005
3. Trouble sleeping or sleeping too much	0.82 (0.74, 0.91)	<0.001	0.85 (0.75, 0.95)	0.005	0.94 (0.83, 1.07)	0.376
4. Feeling tired or having little energy	0.68 (0.62, 0.76)	<0.001	0.68 (0.61, 0.76)	<0.001	0.73 (0.64, 0.83)	<0.001
5. Poor appetite or overeating	0.47 (0.41, 0.54)	<0.001	0.58 (0.50, 0.67)	<0.001	0.75 (0.64, 0.88)	0.001
6. Feeling bad about yourself	0.68 (0.59, 0.79)	<0.001	0.72 (0.61, 0.85)	<0.001	0.85 (0.71, 1.02)	0.074
7. Trouble concentrating on things	0.79 (0.69, 0.91)	0.001	0.84 (0.72, 0.97)	0.021	0.88 (0.74, 1.04)	0.140
8. Moving or speaking slowly or too fast	0.64 (0.53, 0.76)	<0.001	0.73 (0.60, 0.89)	0.002	0.87 (0.70, 1.08)	0.270
9. Thought you would be better off dead	0.57 (0.44, 0.75)	<0.001	0.64 (0.47, 0.88)	0.005	0.77 (0.55, 1.10)	0.148

Model 1: no covariates were adjusted. Model 2: adjust for age, sex and race/ethnicity category. Model 3: adjust for age, sex, race/ethnicity, education level, marital status, BMI, smoking status, diabetes, hypertension, CVD, cancer, blood urea nitrogen, cholesterol, total protein, triglycerides, uric acid, creatinine. BMI, body mass index; CVD, cardiovascular disease; PHQ-9, patient health questionnaire 9; AGR, albumin to globulin ratio.

## 4 Discussion

In this study, we observed for the first time a significant negative correlation between AGR levels and depression among U.S. adults. After adjusting for potential confounders, higher AGR levels remained associated with a lower risk of depression. This correlation remained significant even after dividing AGR into quartiles for analysis. Sensitivity analyses and subgroup analyses further supported the robustness of the results. Overall, these findings provide a new perspective on the relationship between AGR and depression, suggesting that AGR may have potential reference value in the research and clinical assessment of depression.

This finding may be closely related to the composition and function of serum proteins because serum proteins composed of albumin and globulin are not only easy to measure but can also accurately reflect the nutritional status and inflammation level of an individual. Albumin is commonly used to assess nutritional status and has recently been considered an important inflammation marker. Studies have shown that albumin synthesis significantly decreases during inflammatory states, possibly due to inflammation factors promoting albumin leakage and the liver prioritizing the synthesis of acute-phase reactants (11, 21). Additionally, albumin has free radical scavenging and antioxidant properties, making it an important non-enzymatic antioxidant for maintaining plasma redox status (6, 22). Lower albumin levels may lead to oxidative stress imbalance, particularly evident in depression patients who usually exhibit higher levels of free radicals and oxidative damage (23). Depression is closely related to immune activation and increased inflammation markers, and albumin levels decrease with inflammation (24). Albumin also carries and transports important metabolites such as fatty acids, magnesium ions, and thyroid hormones, indirectly influencing the occurrence of depression (25–28). Furthermore, lower albumin levels can reduce the utilization of tryptophan, a key amino acid, affecting serotonin production, which is crucial for the pathophysiology of depression (29). Hypoalbuminemia is not only a marker of malnutrition but also negatively impacts multiple organs and systems (30). Evidence indicates that low albumin levels are linked to a heightened risk of cardiovascular disease, which is closely related to depression incidence (31, 32). Previous literature suggests a negative association between albumin levels and depression (33). Multiple studies reveal that albumin quantities in depression patients are significantly decreased relative to normal control groups (34, 35). After antidepressant treatment, albumin levels gradually increase in depression patients (23). However, lower albumin concentrations during remission may increase the risk of relapse (36).

Globulin represents a category of proteins generated by the liver and the immune system (notably plasma cells), including immunoglobulins, complements, and acute-phase reactants, and plays a significant role in immune and inflammatory responses (37). Globulin mainly consists of α1, α2, β, and γ components, and elevated levels usually indicate chronic or acute inflammation. In depression patients, α1 and α2 globulin levels in serum are significantly elevated (38). Additionally, studies have found that depression patients have lower total serum protein and albumin levels, while globulin levels are higher, possibly reflecting abnormal protein metabolism in these patients (39). The results imply that low albumin and elevated globulin have a connection with depression. As a result, decreased AGR levels may be related to depression.

While changes in albumin and globulin levels reflect inflammation and nutritional status and are related to the pathophysiological mechanisms of depression, the predictive effects of single indicators are susceptible to factors such as dehydration, fluid retention, tissue edema, synthesis material deficiency, and liver dysfunction. However, AGR can significantly reduce these interferences. AGR combines the advantages of albumin and globulin, and its imbalance indicates the presence of infection, malnutrition, chronic systemic inflammation, liver function impairment, or autoimmune issues (40). Previous studies have assessed AGR’s prognostic role in many inflammatory, autoimmune, and malignant diseases (41, 42). However, we did not find studies evaluating the relationship between AGR and depression. Our study is the first to show a negative association between AGR and depression.

Through our subgroup analysis, we discovered that the negative relationship between AGR and depression symptoms persisted across diverse demographic and clinical categories, including age, gender, race, marital status, BMI, smoking status, diabetes, and hypertension. This consistency suggests a strong association that is not significantly altered by these variables. However, we found significant interactions within the diabetes and hypertension subgroups, with stronger associations in diabetes patients without hypertension. Regarding diabetes’s influence on the AGR-depression association, the main considerations are related to inflammation: on one hand, diabetes is a metabolic disease characterized by hyperglycemia, leading to chronic inflammation in multiple systems over time. On the other hand, diabetes may affect nutrient absorption and metabolism, leading to malnutrition and subsequently impacting albumin levels. Lower albumin levels may reflect overall deteriorating nutritional status, which is associated with an increased risk of depression.

Although this finding needs further confirmation, lower AGR levels suggest that malnutrition or high inflammation levels may be related to depression risk. While high baseline inflammation and malnutrition alone cannot cause depression, they are significant risk factors. These factors can increase depression risk by affecting brain chemicals, the immune system, and overall physical health. Moreover, higher AGR indicates higher albumin levels and lower globulin levels. During inflammation, decreased albumin and increased globulin play important roles. Elevated AGR is associated with greater gray matter volume in both the olfactory cortex and the parahippocampal gyrus (43), whereas depression patients often exhibit olfactory dysfunction, possibly related to reduced gray matter volume in the olfactory cortex. Additionally, depression patients typically have reduced gray matter volume in the parahippocampal gyrus, related to cognitive dysfunction and emotional processing issues. Recent studies suggest that high AGR helps protect the nervous system and improve cognitive function in elderly people in Japanese communities (44). Furthermore, depression may involve interactions between the immune and hormonal systems. Lower AGR levels may reflect such interaction imbalances, such as lower albumin levels being related to immune and hormonal imbalances in the body, exacerbating depression symptoms (45).

In this study, we used the PHQ-9 scale to assess participants’ depression symptom severity. Results showed that participants with higher AGR levels scored lower on PHQ-9 questions 1, 2, 4, and 5, indicating better mental health. Higher AGR levels made participants more interested in activities, had higher, happier, or more hopeful moods, felt energetic and vibrant, and maintained a balanced diet.

Currently, the diagnosis of depression mainly relies on patients’ self-reports and clinicians’ subjective judgments, an approach that may lead to the risk of underestimating symptoms or delaying intervention (46). If AGR is included in depression screening programs, it may be able to help medical staff more accurately identify high-risk groups and may help to take corresponding intervention measures in a more timely manner. Given the easy availability and low cost of AGR, its application prospects as a potential biomarker for depression may be broad, especially in the early detection and optimization of intervention strategies, and may have important potential.

However, although this study found a significant negative correlation between AGR and depression risk, this correlation does not necessarily imply a direct causal relationship. Therefore, in clinical practice, although AGR might be used as a reference indicator to assess the risk of depression, especially in patients with chronic inflammation or malnutrition, clinicians still need to interpret this indicator with caution. Specifically, changes in AGR levels may reflect a patient’s overall health, such as inflammation levels and nutritional status, but they cannot alone be used to diagnose depression.

When formulating treatment plans, AGR should be considered in conjunction with other clinical indicators to avoid over-reliance on a single biomarker. Although the correlation of AGR suggests its potential in identifying people at high risk of depression, further research is needed to determine its applicability in different populations and to explore its specific application value in depression management. Our study has several strengths: we systematically studied the relationship between AGR and depression symptoms for the first time, filling a gap in the existing literature. Additionally, the study used a representative and large-scale US population sample and employed a complex multi-stage probability sampling design, strengthening the validity and applicability of the results.

That said, our research is subject to certain limitations: first, as the research employs a cross-sectional design, definitive causality between AGR and depression symptoms cannot be inferred. Future studies should adopt prospective designs to obtain more robust causal evidence. Second, our findings are only applicable to the US population, limiting their generalizability to other populations. Additionally, since this study only included participants with relatively complete data and excluded those with incomplete data, this have introduced selection bias, especially if these exclusions are potentially associated with variables like depression or AGR. Moreover, depression diagnosis relies on participants’ self-reports, which lead to recall bias. Future research should adopt more rigorous survey methods and clinical diagnostic tools to more accurately assess depression symptoms. Lastly, although we adjusted for multiple potential confounders, residual confounding factors still exist.

Therefore, although this study proposed the negative correlation between AGR and depression for the first time and provided preliminary evidence for its use as a potential biomarker, further longitudinal studies and multicenter clinical trials are still needed to verify its causal relationship and specificity. In addition, the practical application value of AGR as a reference indicator in depression screening and treatment also needs to be explored in large-scale studies.

## 5 Conclusion

In conclusion, the NHANES data-based study reveals a significant association between higher AGR levels and a reduced risk of depression in a representative US adult population. This finding emphasizes the correlation between AGR levels and depression, suggesting the potential of AGR as a biomarker for identifying individuals who may be at a higher risk of depression. The exact mechanisms linking AGR to depression are not yet fully understood, and our study does not establish causality. Future research should continue to investigate these correlations to clarify the underlying mechanisms. Understanding these relationships will be important for assessing their relevance in clinical settings and for guiding future intervention strategies based on robust scientific evidence.

## Data Availability

The original contributions presented in the study are included in the article/Supplementary material, further inquiries can be directed to the corresponding author.
