# Supplementary material for: A study investigating how the albumin-globulin ratio relates to depression risk within U.S. adults: a cross-sectional analysis
Source: Front Nutr. 2024 Oct 3;11:1453044. doi: 10.3389/fnut.2024.1453044 (PMC11484099; doi:10.3389/fnut.2024.1453044)
Supplement: Supplementary file 1 [file Table_1.DOCX]

Supplementary Material

**Supplementary Table1**  Baseline characteristics of participants by AGR quartiles in NHANES 2005–2018, weighted.

| Characteristics | Serum albumin to globulin ratio quartiles | | | | | p value |
| --- | --- | --- | --- | --- | --- | --- |
|  | Total | Q1  (<1.29) | Q2 (1.29-1.46) | Q3 (1.47-1.66) | Q4 (>1.66) |  |
| Number of participants | 31363 | 7661 | 7873 | 7874 | 7955 |  |
| Age, years | 47.71 ± 16.82 | 49.01 ± 16.73 | 48.43 ± 16.79 | 47.36 ± 16.80 | 46.76 ± 16.85 | <0.001 |
| Sex, n (%) |  |  |  |  |  | <0.001 |
| Male | 15661(49.32) | 2838(34.45) | 3558(41.45) | 4277(52.41) | 4988(60.63) |  |
| Female | 15702(50.68) | 4823(65.55) | 4315(58.55) | 3597(47.59) | 2967(39.37) |  |
| Race/ethnicity, n (%) |  |  |  |  |  | <0.001 |
| Mexican American | 4929(8.34) | 1383(11.91) | 1431(10.23) | 1256(8.40) | 859(4.96) |  |
| Other Hispanic | 3015(5.42) | 807(7.69) | 819(6.24) | 780(5.43) | 609(3.58) |  |
| Non-Hispanic white | 13662(68.71) | 1944(47.67) | 2988(63.11) | 3749(71.13) | 4981(82.40) |  |
| Non-Hispanic Black | 6477(10.41) | 2771(24.23) | 1800(12.82) | 1202(7.30) | 704(3.56) |  |
| Other races | 3280(7.12) | 756(8.51) | 835(7.59) | 887(7.74) | 802(5.49) |  |
| Education level, n (%) |  |  |  |  |  | <0.001 |
| Less than high school | 7530(15.31) | 2286(21.28) | 2042(16.97) | 1812(15.03) | 1390(11.05) |  |
| High school or GED | 7241(23.34) | 1826(26.38) | 1803(23.82) | 1859(23.69) | 1753(21.02) |  |
| College or above | 16592(61.34) | 3549(52.35) | 4028(59.21) | 4203(61.28) | 4812(67.93) |  |
| Marital status, n (%) |  |  |  |  |  | <0.001 |
| Never married | 5593(17.62) | 1409(18.66) | 1332(17.36) | 1358(16.47) | 1494(18.16) |  |
| Married/living with partner | 18784(63.91) | 4114(58.15) | 4672(62.40) | 4902(65.20) | 5096(67.14) |  |
| Separated/divorced/widowed | 6986(18.47) | 2138(23.19) | 1869(20.24) | 1614(18.33) | 1365(14.70) |  |
| BMI (kg/m^2^), n (%) |  |  |  |  |  | <0.001 |
| <25.0 | 8848(29.46) | 1451(19.12) | 1929(24.63) | 2457(30.51) | 3011(37.77) |  |
| 25.0–29.9 | 10436(33.04) | 2141(26.30) | 2596(31.61) | 2779(34.72) | 2920(36.42) |  |
| ≥30 | 12079(37.50) | 4069(54.58) | 3348(43.76) | 2638(34.77) | 2024(25.80) |  |
| Smoking status, n (%) |  |  |  |  |  | <0.001 |
| Never | 17141(54.57) | 4447(57.79) | 4478(57.12) | 4167(52.53) | 4049(52.66) |  |
| Former | 7737(25.23) | 1761(22.72) | 1848(23.86) | 1979(26.04) | 2149(26.94) |  |
| Current | 6485(20.19) | 1453(19.48) | 1547(19.02) | 1728(21.43) | 1757(20.40) |  |
| Diabetes, n (%) |  |  |  |  |  | <0.001 |
| Yes | 4081(9.56) | 1476(16.18) | 1101(10.70) | 841(8.24) | 663(6.15) |  |
| No | 25436(84.55) | 5650(76.61) | 6274(82.48) | 6602(86.16) | 6910(89.11) |  |
| Borderline | 1846(5.89) | 535(7.21) | 498(6.81) | 431(5.60) | 382(4.74) |  |
| Hypertension, n (%) |  |  |  |  |  | <0.001 |
| Yes | 14083(39.67) | 4112(48.36) | 3592(41.03) | 3302(38.67) | 3077(34.68) |  |
| No | 17280(60.33) | 3549(51.64) | 4281(58.97) | 4572(61.33) | 4878(65.32) |  |
| CVD, n (%) |  |  |  |  |  | <0.001 |
| Yes | 2680(6.83) | 826(9.77) | 682(7.04) | 594(6.42) | 578(5.37) |  |
| No | 28683(93.17) | 6835(90.23) | 7191(92.96) | 7280(93.58) | 7377(94.63) |  |
| Cancer, n (%) |  |  |  |  |  | <0.001 |
| Yes | 2993(10.16) | 682(9.31) | 703(9.64) | 725(9.73) | 883(11.35) |  |
| No | 28370(89.84) | 6979(90.69) | 7170(90.36) | 7149(90.27) | 7072(88.65) |  |
| Depression, n (%) |  |  |  |  |  | <0.001 |
| Yes | 2755(7.66) | 856(11.38) | 748(8.47) | 619(7.09) | 532(5.48) |  |
| No | 28608(92.34) | 6805(88.62) | 7125(91.53) | 7255(92.91) | 7423(94.52) |  |
| PHQ-9 scores | 3.01 ± 4.05 | 3.73 ± 4.64 | 3.21 ± 4.16 | 2.87 ± 3.95 | 2.58 ± 3.61 | <0.001 |
| Albumin, g/dL | 4.26 ± 0.33 | 3.91 ± 0.29 | 4.15 ± 0.24 | 4.32 ± 0.24 | 4.50 ± 0.26 | <0.001 |
| Globulin, g/dL | 2.83 ± 0.43 | 3.44 ± 0.36 | 3.01 ± 0.19 | 2.77 ± 0.17 | 2.42 ± 0.23 | <0.001 |
| Blood urea nitrogen mg/dL | 13.66 ± 5.36 | 13.39 ± 6.65 | 13.56 ± 5.37 | 13.61 ± 4.96 | 13.92 ± 4.84 | <0.001 |
| Cholesterol, mg/dL | 194.52 ± 41.23 | 191.80 ± 41.53 | 196.44 ± 40.87 | 195.88 ± 41.29 | 193.55 ± 41.17 | <0.001 |
| Total protein, g/dL | 7.10 ± 0.45 | 7.36 ± 0.49 | 7.16 ± 0.41 | 7.09 ± 0.39 | 6.92 ± 0.40 | <0.001 |
| Triglycerides, mg/dL | 152.22 ± 120.44 | 150.81 ± 115.56 | 153.86 ± 112.15 | 152.74 ± 115.59 | 151.43 ± 132.11 | 0.440 |
| Uric acid, mg/dL | 5.44 ± 1.40 | 5.50 ± 1.50 | 5.41 ± 1.45 | 5.46 ± 1.39 | 5.41 ± 1.32 | <0.001 |
| Creatinine, mg/dL | 0.89 ± 0.36 | 0.90 ± 0.61 | 0.87 ± 0.32 | 0.89 ± 0.25 | 0.91 ± 0.23 | <0.001 |

Mean ± SD for weighted continuous variables; % for weighted categorical variables.

Abbreviations: BMI, body mass index; CVD, cardiovascular disease; PHQ-9, patient health questionnaire 9; AGR, albumin to globulin ratio.

**
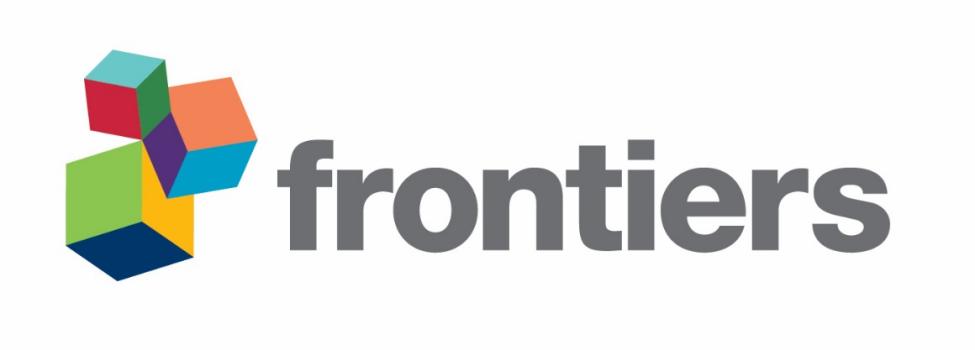
**

**Supplementary Figure 1.** The figure legends are required to have the same font as the main text, 12 point normal Times New Roman, single spaced. Please use a single paragraph for each legend and prepare the figures keeping in mind the PDF layout.
